# Interstitial Lung Diseases in Developing Countries

**DOI:** 10.5334/aogh.2414

**Published:** 2019-01-22

**Authors:** Pilar Rivera-Ortega, Maria Molina-Molina

**Affiliations:** 1 Interstitial Lung Disease Unit, Pulmonology Service, Bellvitge University Hospital. Instituto de Investigación Biomédica de Bellvitge (IDIBELL), ES; 2 Centro de Investigación Biomédica en Red de Respiratorio (CIBERES), Barcelona, ES

## Abstract

More than 100 different conditions are grouped under the term interstitial lung disease (ILD). A diagnosis of an ILD primarily relies on a combination of clinical, radiological, and pathological criteria, which should be evaluated by a multidisciplinary team of specialists. Multiple factors, such as environmental and occupational exposures, infections, drugs, radiation, and genetic predisposition have been implicated in the pathogenesis of these conditions. Asbestosis and other pneumoconiosis, hypersensitivity pneumonitis (HP), chronic beryllium disease, and smoking-related ILD are specifically linked to inhalational exposure of environmental agents. The recent Global Burden of Disease Study reported that ILD rank 40th in relation to global years of life lost in 2013, which represents an increase of 86% compared to 1990. Idiopathic pulmonary fibrosis (IPF) is the prototype of fibrotic ILD. A recent study from the United States reported that the incidence and prevalence of IPF are 14.6 per 100,000 person-years and 58.7 per 100,000 persons, respectively. These data suggests that, in large populated areas such as Brazil, Russia, India, and China (the BRIC region), there may be approximately 2 million people living with IPF. However, studies from South America found much lower rates (0.4–1.2 cases per 100,000 per year). Limited access to high-resolution computed tomography and spirometry or to multidisciplinary teams for accurate diagnosis and optimal treatment are common challenges to the management of ILD in developing countries.

## Introduction

**Figure 1:**
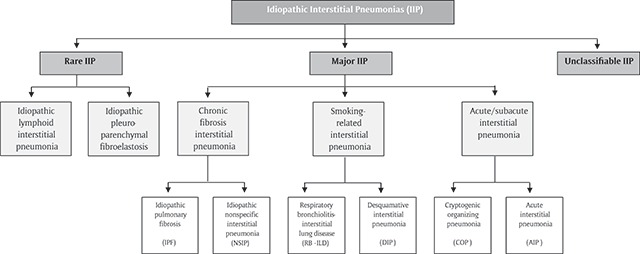
Classification of Idiopathic Interstitial Pneumonias. IIP: Idiopathic Interstitial Pneumonia. Adapted from Am J Respir Crit Care Med. 2013, Vol 188, Iss. 6, pp. 733–748 [2].

Interstitial lung diseases (ILD) are more than 100 pulmonary conditions that affect the alveolar structures, pulmonary interstitium, and/or small airways. A diagnosis of ILD relies on the combination of clinical, radiological, and pathological criteria. Among ILD, the most prevalent are idiopathic pulmonary fibrosis (IPF), sarcoidosis, hypersensitivity pneumonitis (HP), ILD as a manifestation of connective tissue disease (CTD), drug-induced ILD, and pneumoconiosis [1,2]. The idiopathic interstitial pneumonias (IIP) are a group of ILD of unknown cause, which are classified in three main entities: major, rare, and unclassifiable IIP (Figure 1) [2]. Overall, only about a third of ILD cases have an identifiable etiology [3].

Multiple factors such as environmental and occupational exposures, infections, drugs, radiation, and genetic predisposition have been implicated in the pathogenesis of ILD [1,4,5,6]. Several studies suggest a rising trend in the worldwide prevalence of ILD; however, rates vary significantly across different geographic areas [7,8,9,10,11,12,13].

## Epidemiology of ILD

Despite being rare diseases, the recent Global Burden of Disease Study reported that, between 1990 and 2013, there was an 86% increase in ILD-related years of life lost (YLL); as a consequence ILD were included, for the first time, among the top 50 causes of global YYL [14].

Most data on the epidemiology of ILD has been derived from prospective series reported by respiratory physicians (Table 1) [15]. One of the first published ILD registries was conducted by Coultas et al. in New Mexico, United States (US), between 1988 and 1990 [7]. In this region, the prevalence of ILD was 20% higher in males (80.9 per 100,000 persons) than in females (67.2 per 100,000 persons). Similarly, the overall incidence of ILD was slightly more common in males (31.5 per 100,000 persons per year) than females (26.1 per 100,000 persons per year). The authors concluded that the occurrence of ILD in the general populations may be more common than previously estimated based on selected populations [7].

**Table 1:** Prevalence and Incidence of Interstitial Lung Diseases in Developed and Developing Countries.

	Developed countries													Developing countries		

	Europe										America		Asia	Asia		Europe/Asia

	Flanders (Belgium) 1992–1996		Germany 1995	Italy 1997–1999	Spain/RENIA 1998–2000	Spain/SEPAR 2000–2001	Greece 2004		Denmark 2003–2009	EXCITING-ILD (Germany) 2014–2016	New Mexico (United States of America) 1988–1990		Saudi Arabia 2008–2011	India 1997	India Registry 2012–2015	Turkey 2007–2009

	Prevalent cases	Incident cases	Incident cases	Prevalent cases	Incident cases	Incident cases	Prevalent cases	Incident cases	Incident cases	Incident cases	Prevalent cases	Incident cases	Incident cases	Incident cases	Incident cases	Incident cases

Subjects	362	264	234	1138	744	511	967	254	431	201	258	202	330	260	1084	2245
*Unknown etiology*																

Sarcoidosis	112 (31)	69 (26)	83 (35)	344 (30)	87 (12)	76 (15)	330 (34)	60 (23)	–	46 (23)	30 (11.6)	16 (7.8)	67 (20)	140 (53.8)	85 (7.8)	771 (37.6)
IPF/IIP*	62 (17)	50 (19)	76 (32)	417 (37)	287 (39)	215 (42)	234 (24)	66 (25)	121 (28)/186 (43)	64 (32)/82 (41)	58 (22.5)	63 (31.2)	77 (23.3)/108 (32.3)	79 (30.4)	148 (13.7)	408 (19.9)/532 (26)
COP-BOOP	10 (2.3)	9 (3.4)	16 (6.8)	57 (5)	38 (5.1)	53 (10)	51 (5.3)	18 (7)	10 (3)	4 (2)	–	1 (0.5)	7 (2.1)	–	–	58 (2.8)
(C)EP	9 (2.2)	7 (2.7)	–	27 (2.3)	–	–	21 (2.2)	7 (2.7)	4 (1)	–	3 (1.2)	1 (0.5)	1 (0.3)	–	–	19 (1)
CTD	27 (7.5)	19 (7.2)	5 (2.1)	–	69 (9.3)	51 (19)	120 (12)	30 (12)	54 (13)	12 (6)	33 (12.8)	18 (9)	115 (34.8)	35 (13.5)	151 (13.9)	201 (9.8)
Vasculitis^#^	5 (1.4)	4 (1.5)	2 (0.8)	25 (2.2)	–	–	14 (1.5)	6 (2.3)	–	–	2 (1.2)	8 (4)	–	–	–	42 (2)
EG-HX	13 (3.6)	7 (2.7)	–	73 (7.2)	6 (0.8)	15 (3)	37 (3.8)	7 (2.7)	8 (2)	–	2 (0.8)	–	1 (0.3)	–	–	28 (1.3)
*Exogenous etiology*																

EAA (HP)	47 (13)	32 (12)	25 (11)	50 (4.3)	38 (5.1)	34 (7)	25 (2.6)	7 (2.7)	32 (7)	36 (18)	–	3 (1.5)	21 (6.4)	–	513 (47.3)	82 (4)
Drug^¶^	12 (3.3)	12 (5)	6 (2.6)	21 (1.8)	–	21 (4)	17 (1.8)	4 (1.5)	20 (5)	4 (2)	6 (2.3)	10 (5)	4 (1.2)	3 (1.2)	–	71 (3.5)
Pneumoconiosis°	19 (5)	18 (6.8)	6 (2.6)	–	55 (7.4)	–	20 (2)	8 (3.1)	–	–	36 (13.9)	21 (10.4)	–	3 (1.2)	–	241 (11.8)
*Variable etiology*																

Non specific fibrosis	33 (9.1)	27 (10)	12 (5.1)	–	69 (9.3)	–	82 (8.5)	40 (15)	62 (14)	12 (6)	43 (16.7)	28 (13.9)	6 (1.8)	–	–	–
Others	13 (3.8)	10 (3.8)	–	124 (11)	76 (10)	9 (2)	15 (1.5)	6 (2.3)	101 (25)	9 (4)	44 (17)	33 (16.2)	5 (1.5)	–	187 (17.3)	58 (2.7)

n: number of subjects. Data are presented as n (%), unless otherwise stated. RENIA: Registry of Interstitial Pneumopathies of Andalusia; SEPAR: Sociedad Española de Neumología y Cirugía Torácica; EXCITING-ILD: Exploring Clinical and Epidemiological Characteristics of Interstitial Lung Diseases; IPF: idiopathic pulmonary fibrosis; IIP: idiopathic interstitial pneumonia; COP: cryptogenic organizing pneumonia; BOOP: bronchiolitis obliterans organizing pneumonia (not necessarily cryptogenic); (C)EP: (chronic) eosinophilic pneumonia; CTD: connective tissue disease; EG: eosinophilic granuloma; HX: histiocytosis X; EAA (HP): extrinsic allergic alveolitis (hypersensitivity pneumonitis). * If there is data available from IIP, it will be shown separately, after the IPF data. The IPF is part of the IIP. ^#^ Goodpasture’s, granulomatosis with polyangiitis (Wegener’s), Chrug-Strauss, etc. ^¶^ Radiation was also included in the Italian, SEPAR, US, India, and Turkey registries. ^°^ Coal worker’s pneumoconiosis was excluded in the Flemish, Italian and SEPAR registries. The American and Turkish registries include occupational exposition. The Indian study (1997) includes only silicosis.

Several European studies have reported on the frequency and distribution of ILD [16,17,18,19,20,21,22]. Studies show that the most frequent ILD are IPF and sarcoidosis, which together comprise about 50% of cases. The data also show considerable variability between countries, such a lower proportion of IPF in Belgium, of sarcoidosis in Spain, of ILD associated with CTD in Germany, and a higher incidence of HP in Germany. A Danish cohort study reported an incidence of ILD and IPF were 4.1 per 100,000 persons per year and 1.3 per 100,000 persons per year, respectively [18]. In a Spanish ILD registry including cases from 23 pulmonary medicine centers, the estimated incidence of ILD was 7.6 per 100,000 persons per year [19]. Finally, in Greece, the ILD incidence rate was estimated to be 4.63 per 100,000 persons per year [21].

Few studies have evaluated the rates of ILD in Asia. An epidemiological study that enrolled 2,245 patients with newly diagnosed ILD from 31 centers in 19 Turkish cities [23] showed an overall incidence of ILDs of 25.8 cases per 100,000 persons. Overall, in 24% of ILD cases a specific etiology could be identified, 39% were granulomatous diseases, 24% were idiopathic, and 4% were unclassified. Sarcoidosis (37%) was the most common disease, whereas cases with IPF constituted 20% of patients. In India, chronic hypersensitivity pneumonitis (cHP), pulmonary fibrosis associated to CTD, sarcoidosis and IPF are the most common entities [24]. A more recent ILD-India registry, which collects data from 27 centers in 19 cities, reported 148 cases (14%) of IPF and 151 cases (14%) of CTD-ILD, among 1,084 patients with ILD [25]. A single-center study of 330 ILD cases from Saudi Arabia found that the most frequent disease was CTD-ILD (35%), followed by IPF (23%) and sarcoidosis (20%) [26].

Less data is available about the prevalence of ILD in Latin America. However, a Mexican study that abstracted cases from the National Database of Mortality showed that fibrotic ILD represents the 0.4% of all registered deaths [27].

## Occupational and Environmental Exposures

ILD have been more closely associated with occupational exposures than any other respiratory disease. Classic examples of occupational diseases are the pneumoconiosis caused by asbestos (asbestosis), silica (silicosis), and coal dust (coal worker’s pneumoconiosis). In 2000 in Europe, it was estimated that a total of 7,200 cases of pneumoconiosis were related to occupational exposures to asbestos, silica, and coal dust [28].

Although individual susceptibility plays a role in mineral pneumoconiosis, they are generally considered to be caused by the progressive accumulation of toxic dust in the lungs. In contrast, individual susceptibility and/or immunological sensitization play a more dominant role in the pathogenesis of ILD such as HP, chronic beryllium disease (berylliosis) or hard metal/cobalt/related lung disease [28].

## Asbestos

The exposure to asbestos is the cause of asbestosis and one of the most common ILD related to occupational exposures. World Health Organization (WHO) officials estimate that 125 million people worldwide are annually exposed to asbestos in occupational settings, and more than 107,000 people die each year and 1,523,000 Disability Adjusted Life Years (DALYs) are attributable to asbestos-related diseases [29].

Asbestos has been banned in most developed countries, but is still used in many developing nations. Many countries are now experiencing an epidemic of asbestos-related disorders as a legacy of occupational exposures in the 1960s–1980s because of the long latency period between exposure and disease onset. Consequently, it is expected that asbestos-related mortality and morbidity will continue to increase. Although the most feared complications of asbestos inhalation are the malignant conditions such as mesothelioma and lung cancer, asbestos exposure more frequently results in benign, but potentially disabling, conditions such as pleural plaques, diffuse pleural thickening, and asbestosis (pulmonary fibrosis) [30].

Because of its durability and tensile strength, asbestos has been used in over 3,000 products. The top asbestos producing countries are Russia, China, and Kazakhstan [31]. Despite European measures to control imports, the global production of asbestos has not decreased [29]. A number of substitutes have replaced asbestos in developed countries, including cellulose polyacrylonitrile, glass fiber, and unplasticised polyvinyl chloride (PVC). Although asbestos substitutes are expensive, they work out to be cheaper in the long run because of their durability [32]. However, as these materials have similar physiochemical properties to asbestos, there is still a concern that some may also cause pulmonary fibrosis [33,34].

Relatively high levels of asbestos inhalation are required to produce asbestosis, although there are reports of asbestosis cases following moderate exposure history [35]. Accepted diagnostic criteria are based on a compatible exposure history with clinical and radiographic features characteristic of asbestosis [36]. Unfortunately, a firm diagnosis may be difficult to establish, as asbestosis resembles a variety of other inflammatory and fibrotic lung diseases such as pneumoconiosis, IPF, respiratory bronchiolitis, and sarcoidosis [30]. The phenomenon of para-occupational or “take home” asbestos exposure due to dust accumulated on the worker’s clothing or hair has been recognized for over 50 years [30]. Multiple ARD cases of ARD caused by para-occupational exposure have been reported in the literature [37,38,39]. However, the vast majority of the cases occurred among family members of workers in industries characterized by high exposures and nearly always to amphibole fibers.

## Other Occupational Exposures

Inorganic dusts are an important cause of pulmonary fibrosis, respiratory disability, and death. Silicosis is a pulmonary disease resulting from the inhalation and accumulation of inorganic silica dust in the lung. The risk of disease is related to lifetime cumulative exposure and to amount of inhaled crystalline silica, which depends on the concentration and the size of breathable particles (<5 um) and on individual susceptibility [40]. Silicosis has a relatively high prevalence among workers involved in mica mining, silica and fire clay brick making, iron and steel foundries, metal casting, grinding, boiler-scaling, and polishing and manufacturing of glass, paints, and rubber [24,41]. Special attention is required for new construction materials, such as “quartz conglomerates,” which contains a high proportion of silica to increase the stiffness and may be inhaled when cutting or polishing it [40]. Although prevention efforts have been in place for many decades, silicosis is a serious problem worldwide, particularly in developing countries, where the burden is often under-reported because of inadequate surveillance [42]. In the Brazilian gold-mining area in Minas Gerais, more than 4,500 workers were reported to have had silicosis between 1978 and 1998 [43]. Of gold miners in South Africa dying from accidents (e.g., injuries, burns, poisoning, and drowning), proportions with silicosis identified at autopsy increased from 3% to 32% for black miners and from 18% to 22% for white miners between 1975 and 2007 [44]. Most recently, exposure to silica in the textile sector has been reported as a novel and unusual source of silicosis in Turkey between 1991 and 2006, as a result of sandblasting denim; in this study, of 145 evaluated workers, 53% were diagnosed with silicosis [45].

Silicosis is also an occupational health concern in developed countries; according to the carcinogen exposure report (CAREX) released in 2000, 3.2 million European workers were exposed to crystalline silica [46]. China has the highest number of cases of silicosis, with more than 500,000 cases recorded between 1991 and 1995, and more than 24,000 deaths annually [42,47]. In the United States, more than 121,000 workers were exposed to breathable crystalline silica in 1993 [48], and 3,600–7,300 silicosis cases occurred annually from 1987 to 1996 [49].

Byssinosis is a chronic respiratory disease observed among workers exposed to cotton, flax, and soft hemp dust. Cotton processing employs many workers throughout the world and carries the maximum risk of byssinosis among those involved in the initial processes of yarn manufacture [50]. At the beginning of the 1990s, byssinosis rates declined in developed countries due to the introduction of dust control measures in the textile mills; however, similar patterns have not yet been observed in developing areas. For example, in India, studies have shown a high prevalence of byssinosis in textile mills [51,52,53]. A study from South Africa that examined 2,411 textile workers showed that the prevalence of byssinosis was highest (44%) among bale opening and blowroom workers [54]. In a study conducted in a textile factory in Cameroon, the overall prevalence of byssinosis was 28% [55]. A study from Ethiopia showed that the prevalence of byssinosis was 43% among blowing workers and 38% in carding workers [56]. Similarly, two studies from Sudan showed a high prevalence of byssinosis (67% and 40%, respectively) in workers in the blowing and carding sections [57,58]. A strong correlation between textile factory site and risk of byssinosis was reported in a study from Egypt, which showed disease in 21% of workers in opening and cleaning sections, 13% of workers in the carding and combing rooms, compared to <3% in other workers [59]. A more recent study in a cotton factory in Benin, found that the prevalence of byssinosis was 21% in exposed compared to 8% in unexposed workers (p = 0.006) [60].

## HP

HP due to organic dust exposures is common and some cases may progress to pulmonary fibrosis. Agents capable of inducing HP are found in the workplace, home, and recreational environments. HP-inducing antigens are commonly classified in five broad categories represented by disease prototypes: bacteria, fungus, mycobacteria, proteins, and chemical products (Table 2) [61,62]. The list of antigens implicated in HP shows the broad spectrum of possible causes and the difficulties to abrogate exposures. The mechanisms leading to acute versus chronic forms of HP after antigen exposure is an unresolved question that has important management and prognostic implications. Chronic HP, which seems to be the consequence of long-term low-level exposure, can clinically resemble IPF and have a similar long-term outcome. Conversely, acute HP, which is usually a consequence of short exposure to high concentrations of an antigen, usually presents an inflammatory pulmonary response.

**Table 2:** Common Types of Hypersensitivity Pneumonitis According to Major Classes of Antigens.

Class of antigens	Specific antigens	Sources	Type of disease

Organic particulate matters			

***Microbes***			
Bacteria	*Saccharopolyspora rectivirgula, Thermoactinomyces vulgaris*	Moldy hay, grain	Farmer’s lung
Fungus	*Aspergillus* species	Moldy hay, grain Moldy compost and mushrooms	Farmer’s lung Mushrooms worker’s lung
	*Trichosporon cutaneum*	Contaminated houses	Japanese summer-type HP
	*Penicillium* species	Moldy cork Moldy cheese or cheese casings	Suberosis Cheese washer’s lung
	*Alternaria* species	Contaminated wood pulp or dust	Woodworker’s lung
Mycobacteria	*Mycobacterium avium-intracellulare*	Mold on ceiling, tub water Mist from pool water, sprays and fountains	Hot tub lung Swimming pool lung
***Proteins***			
Animal proteins	Proteins in avian droppings and serum and on feathers	Parakeets, budgerigars, pigeons, parrots, cockatiels, ducks	Pigeon breeder’s lung, bird fancier’s lung
	Avian proteins	Feather beds, pillow, duvets	Feather duvet lung
	Silkworm proteins	Dust from silkworm larvae and cocoons	Silk production HP
Plant’s proteins	Grain flour (wheat, rye, oats, maize)	Flour dust	Flour dust alveolitis
	Legumes (soy)	Legumes (soy), flour dust	Soya dust alveolitis
	Wood (cabreuva, cedar, mahagony, pine, ramin, umbrella pine)	Wood particles	Wood fiber alveolitis
**Inorganic particulate matters**			

Chemicals products	Diisocyanates, trimellitic anhydride	Polyurethane foams, spray paints, dyes, glues	Chemical worker’s lung

HP: hypersensitivity pneumonitis.

## Exposure to Air Pollution

Air pollution is a well-established risk factor for airway diseases and lung cancer. However, few studies have investigated the relationship between air pollution and ILD [63]. Ambient air pollution includes chemical, biologic, and particulate materials released into the atmosphere. Of the air pollutants regulated by the United Sates Environmental Protection Agency (particulate matter [PM], ozone [O_3_], nitrogen dioxide [NO_2_], sulfur dioxide, carbon monoxide, and lead), PM, ground-level O_3_, and NO_2_ have been most strongly associated with adverse respiratory outcomes. PM is a uniquely complex mixture that may include solid particles, liquids, and vapors. Sources of PM include geologic formations (e.g., sand, salt), metals, and fossil fuel combustion (e.g., diesel exhaust particles, black carbon). PM is typically defined by size, such as PM ≤ 10 um or ≤ 2.5 um in aerodynamic diameter (PM_10_ and PM_2.5_, respectively); however, its toxicity varies depending on factors like particle weight and composition, as well as host factors determining the location and density of deposition in the respiratory tract [64]. Organic components of PM may trigger abnormal immune responses leading to inflammation, epithelial damage, and over time, fibrosis [63]. NO_2_ is emitted whenever fossil fuels are combusted; it is a good marker of traffic-related air pollution and is an indicator for the larger group of nitrogen oxides (NO_x_). NO_x_ combine with other compounds, such ammonia and moisture, to form small particles capable of penetrating deep into the lung. Tropospheric O_3_ exists within 10 km of the Earth’s surface and is photochemically produced through the reactions of sunlight with other pollutants like volatile organic compounds and NO_x_. In both human and animal studies, O_3_ has been found to induce airway hyperreactivity and airway inflammation, as well as to modify the cell/surface phenotypic expression of immunoregulatory proteins [63,65,66,67,68].

**Figure 2:**
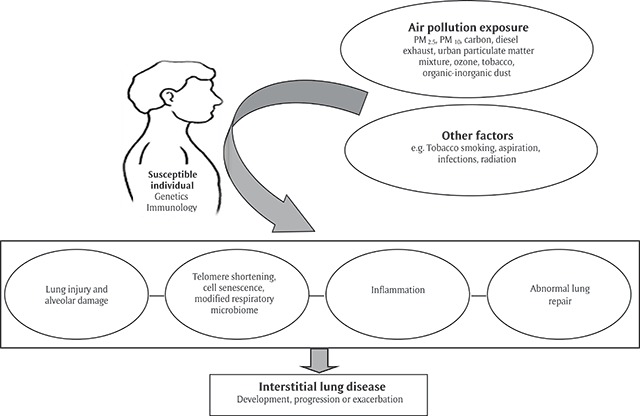
Mechanisms by which Air Pollution Exposure Could Trigger Intertidal Lung Diseases. Modified from Chest 2015;147(4):1161–1167 [63]. PM_2.5_: particulate matter <2.5 um in aerodynamic diameter; PM_10_: particulate matter <10 um in aerodynamic diameter.

Air pollution could be associated with the development, progression, or exacerbation of ILD via mechanisms of lung injury-alveolar damage [69,70], telomere shortening [71,72,73,74], cell senescence [75], changes in the respiratory microbiome [76,77,78,79], inflammation [80,81,82], and/or abnormal lung repair (Figure 2) [63,83,84,85,86]. Additionally, individual genetic or epigenetic factors may impact the phenotypic expression of ILD resulting from environmental exposures, and future research is necessary to delineate these mechanisms [63].

According to the recent WHO report, air pollution levels in urban areas increased during 2008 and 2013 by 8%. High-income regions of the Americas, Europe, and the Western Pacific demonstrate decreasing air pollution, while the other developing countries had increasing levels [87].

## ILD Associated with CTD

The intersection of CTD and the ILD is complex. Although often considered as a single entity, “CTD-ILD” actually reflects a heterogeneous spectrum of diverse CTD and a variety of patterns of interstitial pneumonia. The evaluation of patients with CTD that develop ILD, or the assessment for underlying CTD in those presenting with presumed “idiopathic” ILD can be challenging and should be optimized by rational immunological testing. When a diagnosis of CTD-ILD is confirmed, careful assessments to determine extra- versus intra-thoracic disease activity, and degrees of impairment are needed [88].

ILD is a major source of morbidity and the leading cause of mortality in patients with CTD [89,90]. Certain CTD are more likely to be associated with ILD (e.g., systemic sclerosis [SSc], idiopathic inflammatory myopathy [IIM] and rheumatoid arthritis [RA]), but all CTD patients are at risk for developing ILD; moreover, ILD may be the first or only manifestation of CTD [91,92].

Pharmacologic intervention with immunosuppression is the mainstay of therapy for all forms of CTD-ILD, which is usually indicated for clinically significant and/or progressive disease. The management of CTD-ILD is not yet evidence based, and there is a critical need for controlled trials. Non-pharmacologic management strategies and addressing comorbidities or aggravating factors should be part of a comprehensive treatment of these patients [88]. Drug development for CTD-ILD is challenging due to their variable presentation, heterogeneous disease course, and substantial mortality [91]. There have been very few randomized controlled trials (RCTs) in CTD-ILD, and further advancements are adversely affected by the lack of well-defined outcome measures [93,94]. In a well-designed RCT of cyclophosphamide versus placebo in SSc-ILD (Scleroderma Lung Study-1), modest changes were observed in lung physiology and patient-reported outcomes [94]. A recent retrospective analysis demonstrated a similar effect of mycophenolate and cyclophosphamide in SSc-ILD [95]. Clinical trials with anti-fibrotic drugs are being initiated and may provide new alternatives for the treatment of these patients.

Pulmonary vasculitis is defined by the involvement of blood vessels of the lung parenchyma either locally or as part of a systemic vasculitis [24]. Vasculitis of infectious etiology are a more common problem in the developing countries. Tuberculosis may involve the vasculature either as endarteritis obliterans affecting vessels surrounded by necrotic granulomatous tissue or as a true immune complex vasculitis [96]. Fungal infections causing angioinvasion (aspergillosis, mucormycosis, and candidiasis) are more often seen in developing countries or in patients on prolonged treatment with steroids or other immunosuppressive drugs [97].

While immunosuppression is the mainstay of therapy for all forms of CTD-ILD, there is limited evidence to support management of these conditions. Non-pharmacologic support and management of comorbidities should be part of a comprehensive treatment plan for individuals with CTD-ILD [88].

## Geoepidemiology of IPF

IPF is the prototype of fibrotic ILDs, a group of pulmonary conditions that do not follow boundaries or geographic preferences. Risk factors for IPF linked to a particular racial group, a specific geographic area or environmental exposure have not been identified. Thus, it is likely that the burden of disease will be concentrated in the most densely populated region of the globe. BRIC countries (Brazil, Russia, India, and China), with an estimated 2.9 billion inhabitants, may comprise 2 million cases of IPF [98,99,100,101,102].

A study using a sensitive diagnostic algorithm found that the incidence and prevalence of IPF in the United States were 14.6 per 100,000 person-years and 58.7 per 100,000 persons, respectively [103]. Another review indicated that the prevalence of IPF in the US and European countries was 14.0–27.9 and 1.25–23.4 cases per 100,000 population, respectively [104]. It is reasonable to assume that variability in age distribution as well as ethnic and genetic differences among the populations may contribute to these findings [105]. While most likely related to differences in access to care, lung transplant databases in 2006 showed that blacks and Hispanics with IPF had a lower survival from time of listing compared with whites [106,107].

Recent data suggest an increasing prevalence and a stable or increasing incidence of IPF in western countries [108,109,110,111,112,113,121,122,123,124]. Incidence and mortality studies from South America suggest a low incidence (0.4–1.2 cases per 100,000 people per year) [114,115]. In a large database Brazilian study, the incidence of IPF was estimated at 0.26 cases per 100,000 persons per year in 1996, rising to 0.48 per 100,000 persons per year in 2010 [114]. The lower incidence in South America may be due to under-diagnosis or under-reporting on death certificates.

There have been few epidemiologic studies in Asian communities. Insurance claims-based studies from East Asia showed a low incidence (1.2–3.8 per 100,000 per year) [116,117,118], although mortality statistics from Japan suggested a higher incidence. In East Asia, the higher severity of disease in study subjects from insurance datasets likely reflects exclusion of milder cases and may explain the lower incidence compared to western countries [116,119]. Adjusted IPF mortality statistics from Oceania ranged from 5.08–6.49 per 100,000 population [120].

## Environment, Smoking, and Diet in IPF

Although “idiopathic” by definition, potential etiological factors have been implicated in the development of IPF [125,126,127]. IPF has been associated with industrial and production-based jobs as well as metal and wood dust occupational exposures [63]. The most well-established environmental risk factor for IPF is tobacco smoking (odds ratio for ever smokers of 1.6, 95% confidence interval [CI]: 1.1–2.4) [128,129,130].

Organic components of PM may trigger abnormal immune responses leading to inflammation, epithelial damage, and over time, fibrosis. There is a small but growing body of evidence suggesting a potential relationship between exposure to air pollution exposure and ILD exacerbations [63]. In a study of 325 patients with IPF, ambient air pollution was found to modify longitudinal changes in lung function, suggesting that pollutants may differentially alter the immunomodulatory pathways associated with IPF [131]. Similarly, a study of a well-defined cohort of patients with IPF found O_3_ and NO_2_ exposure to be associated with an increased risk of acute exacerbation and mortality [132].

Evidence linking diet to IPF is limited. Lungs from patients with IPF appear to be deficient in glutathione [127], suggesting suboptimal antioxidant defenses. High intake of vegetables, green tea, and fish has been associated with a decreased risk for IPF, possibly due to their anti-oxidant properties [133]. Further studies are needed to clarify these findings. Other etiologies may also be implicated in the development of IPF including viral infections, especially hepatitis C and the Epstein-Barr virus. Britton and colleagues demonstrated an increased risk of IPF with the use of antidepressant medications [107,127]; further studies in animal models are necessary to better understand this possible relationship.

## Gender and IPF

Clinical studies in IPF have enrolled a larger proportion of men than women; few studies explicitly report that IPF is more common in men. A study assessing IPF and chronic obstructive pulmonary disease (COPD) showed a significant association with male gender and increased prevalence of combined pulmonary fibrosis and emphysema (CPFE). The OR for male gender having CPFE was 18 (95% CI: 3–773), and subjects with CPFE had a lower median survival time compared to IPF subjects, though this appears to be related to presence of pulmonary hypertension or more severe restrictive lung physiology [134].

## Genetics of Pulmonary Fibrosis

Many clinical disorders that are associated with pulmonary fibrosis have been linked to specific inherited gene mutations and polymorphisms [135,136,137]. Early studies that identified evidence of inherited risk for developing pulmonary fibrosis focused on familial cases, including variants such as genes coding for mucin 5B surfactant proteins [136] or those involved in telomere homeostasis and function [73]. Studies that have focused particularly on genome-wide linkage analyses have identified numerous gene polymorphisms that are associated with increased risk for pulmonary fibrosis [135,138,139,140]. However, not all races have been evaluated, even for widely studied genetic mutations that have demonstrated their association with pulmonary fibrosis. Therefore, global collaboration for genetic studies is a priority to better understand the disease.

## Comorbidities of IPF

IPF is associated with pulmonary or extrapulmonary comorbidities. Pulmonary comorbidities include pulmonary hypertension, emphysema, and lung cancer, while non-pulmonary conditions include venous thromboembolism, coronary artery disease, congestive heart failure, sleep-disordered breathing, gastro-oesophageal reflux disease, and anxiety or depression. Although some of these comorbid conditions share risk factors with IPF, the risk in patients with IPF is still greater than expected by chance. This might indicate that IPF fosters an environment for the development or perpetuation of comorbid conditions, or alternatively that they share unknown causative factors. Optimal management of IPF therefore requires a comprehensive approach, including the identification and treatment of comorbid conditions to optimize patient outcomes [141].

## Current Diagnosis Criteria and Treatment of IPF

In 2011, American Thoracic Society (ATS), European Respiratory Society (ERS), the Japanese Respiratory Society (JRS), and the Latin-American Thoracic Society (ALAT) jointly published an evidence-based statement for the diagnosis and management of IPF [128]. This document provided an update of the diagnosis criteria: [4] 1) exclusion of other known causes of ILD (e.g., domestic and occupational environmental exposures, CTD and drug toxicity); 2) presence of an usual interstitial pneumonia (UIP) pattern on chest high-resolution computed tomography (HRCT); and 3) specific combinations of HRCT and biopsy UIP patterns in individuals undergoing surgical lung biopsy (SLB) [108]. The criteria originated from the evidence that in an appropriate clinical setting, the presence of a classical UIP pattern on the HRCT has a very high positive predictive value (90% to 100%) for a histological diagnosis of UIP [142,143].

In 2015, recommendations for the treatment of IPF were updated based of new scientific evidence [128,144]. This was a major milestone, as for the first time a therapeutic recommendation with a high level of evidence was established for two antifibrotic drugs: Pirfenidone and Nintedanib [145]. These new drugs provide benefits in terms of a significant reduction in mortality, positioning IPF as one of the few areas in respiratory medicine in which treatment could provide such clinically significant improvements [146].

## Conclusions

ILDs are an heterogeneous group of relatively uncommon diseases. Few data are available on ILD epidemiology, especially in developing countries, although the prevalence and incidence seem to be increasing in many areas. IPF is the most common and studied of the idiopathic ILDs, with updated guidelines for diagnosis and new treatment options. The involvement of centers in developing countries should be encouraged, for example through the ILD global registries and/or increased access to expert multidisciplinary team consensus, as it would help to obtain an accurate and prompt diagnosis and health access to treatment. Additionally, these strategies would allow understanding racial and environmental risk factors, and therefore, provide insights in the pathogenesis of ILDs.
